# Patient-reported Outcome Measures in Head and Neck Reconstruction: A Systematic Review Across Disciplines and Geographical Locations

**DOI:** 10.1097/GOX.0000000000007293

**Published:** 2025-12-09

**Authors:** Pablo Pfister, Julia Stoffel, Marie Perrin, Christian Appenzeller-Herzog, Florian S. Halbeisen, Lukas Seifert, Daniel Bodmer, Laurent Muller, Maximilian Burger, Rene D. Largo, Tarek Ismail, Elisabeth A. Kappos

**Affiliations:** From the * Department of Plastic, Reconstructive, Aesthetic and Hand Surgery, University Hospital Basel, Basel, Switzerland; † Faculty of Medicine, University of Basel, Basel, Switzerland; ‡ Faculty of Medicine, University Medical Library, University of Basel, Basel, Switzerland; § Surgical Outcome Research Center, Department of Clinical Research, University of Basel c/o University Hospital of Basel, Basel, Switzerland; ¶ Department of Oral and Cranio-Maxillofacial Surgery, University Hospital Basel, Basel, Switzerland; ∥ Department of Otolaryngology, Head and Neck Surgery, University Hospital Basel, Basel, Switzerland; ** Department of Plastic Surgery, The University of Texas MD Anderson Cancer Center, Houston, TX.

## Abstract

**Background:**

Patient-reported outcome measures (PROMs) have become an integral part of outcome evaluation in reconstructive microsurgery. This study explored the usage of PROMs in microsurgical oncological head and neck reconstruction across surgical specialties and geographic regions.

**Methods:**

A systematic literature search was conducted in Embase, MEDLINE, and Web of Science to identify studies reporting on adult patients undergoing oncological head and neck free flap reconstruction. Data extracted included PROM tools used, study demographics, surgical disciplines involved, procedural details, and outcomes.

**Results:**

Of 5015 screened studies, 354 (n = 30,369 patients) met the inclusion criteria. A total of 94 PROM tools were identified. The most frequently used were the University of Washington Quality of Life Questionnaire (34.75%), nonvalidated tools (25.99%), and the European Organisation for Research and Treatment of Cancer Quality of Life Questionnaire – Head and Neck Module 35 (22.32%). Donor site–specific PROMs were infrequently used (22%), though more frequently used when plastic and reconstructive surgeons were involved (31.11% versus 15.53%, *P* = 0.019). PROM selection varied significantly by specialty and geographic location, and only 20% of studies were interdisciplinary.

**Conclusions:**

Substantial heterogeneity exists in the use of PROMs in head and neck reconstruction, with variations driven by specialty and region. This lack of standardization hinders meaningful cross-study comparisons and the development of robust quality benchmarks. A unified, validated PROM framework is urgently needed to support international evidence-based practice. Future efforts should prioritize interdisciplinary collaboration and incorporate donor-site evaluation to comprehensively assess outcomes in oncological head and neck reconstruction.

**Takeaways**

**Question:** Which patient-reported outcome measures (PROMs) are currently used in microsurgical head and neck reconstruction, and do geographic and interdisciplinary variations exist?

**Findings:** A total of 94 PROM tools were identified, with variation by surgical specialty and geographic location. The most frequently used tools were the University of Washington Quality of Life Questionnaire (34.75%), nonvalidated tools (25.99%), and the EORTC QLQ-H&N35 (22.32%).

**Meaning:** Significant variation in PROM use across specialties and regions limits cross-study comparisons, highlighting the need for a standardized evaluation to improve head and neck reconstruction outcomes.

## INTRODUCTION

Head and neck cancers (HNCs) are the seventh most common malignancies worldwide, with more than 890,000 new cases and 450,000 deaths annually.^1,2^ Over the past 3 decades, the landscape of the HNC population has shifted significantly. Although the incidence of tobacco- and alcohol-related squamous cell carcinomas has steadily declined, there has been a notable rise in cases linked to human papillomavirus.^3^ Concurrently, improved survival rates have led to more survivors seeking optimal functional and aesthetic outcomes.^4^ This is particularly evident in the context of a younger patient population, where the balance between quality and quantity of life increasingly shifts toward prioritizing functional well-being beyond mere cancer survival.^5^

This trend extends into the operating room, where ablative procedures remain essential for ensuring optimal cancer survival, whereas reconstructive procedures are gaining increasing importance. Unlike ablative surgeons, who evaluate outcomes based on survival and cancer recurrence, reconstructive surgeons face challenges in assessing reconstructive success beyond cancer survival.^6,7^ Previously, reported outcomes often focused on factors that may not accurately reflect the patient’s long-term quality of life (QoL) or improvements in patient-centered outcomes, such as operation duration or the risk of reoperation.^6^ It is arguable that patient-reported outcome measures (PROMs) and QoL are the most crucial indicators when evaluating the success of a reconstruction. Beyond facing a life-threatening illness, HNC patients endure treatment-related side effects that impact their functional and psychosocial well-being.^8^ Because HNC affects critical structures responsible for chewing, swallowing, breathing, speech, and facial expression, patients often experience morbidities such as xerostomia, dysphagia, trismus, and malnutrition.^9,10^ Changes in voice and speech can further reduce self-esteem, heighten social anxiety, and contribute to elevated depression rates (ranging from 20% to 40%), which align with HNC’s distinction as having the highest suicide rates among cancer patients.^11–13^ This growing recognition of the profound impact on QoL has driven increased research efforts aimed at improving these outcomes.^14^

PROMs can be used to measure the impact of a disease and its treatment on the patients’ health-related QoL.^15^ PROMs are standardized, validated survey tools that capture patients’ reports of their health outcomes by measuring their symptoms, perception of health status, and/or functional well-being.^16^ Within the scope of HNC, PROMs may be used to assess the impact of individual treatment modalities, such as radiotherapy, chemotherapy, and surgery, as well as to assess the success of reconstructive surgery in restoring function and QoL after cancer treatment.

Despite their increasing relevance, the routine use of PROMs in oncological head and neck reconstruction remains inconsistent. A standardized, discipline-spanning framework for PROM application is lacking, which hinders outcome comparison and evidence-based decision-making.^17^ This study aimed to systematically review the PROM tools currently used in microsurgical head and neck reconstruction. We also examined how PROM selection is influenced by surgical specialty, geographic location, and interdisciplinary collaboration, with the goal of identifying barriers to standardization and highlighting opportunities to improve global practice.

## METHODS

The Preferred Reporting Items for Systematic Reviews and Meta-Analyses statement was used to guide the reporting for this systematic review.^18^ This systematic review was registered in the PROSPERO International Prospective Register of Systematic Reviews (registration date March 6, 2024; ID CRD42024517104).

### Literature Search Strategy

A systematic literature search was performed to identify articles on postoncological head and neck reconstruction in adults, which reported a QoL outcome such as patient satisfaction or a PROM tool assessment. Search strategies for Embase (embase.com), MEDLINE (Ovid), and the Web of Science Core Collection (webofscience.com) were composed by an information specialist (C.A.-H.). They comprised database-specific subject headings and text words covering the search concepts of HNC, surgical flaps, and QoL/PROM. Names of known PROMs were omitted from the search string to prevent bias in retrieval. No restrictions on language or publication date were applied. Conference abstracts were excluded. The year 1980 was selected to ensure the inclusion of early foundational studies and to capture the full evolution of PROM development in head and neck reconstruction. Search syntax was translated from Embase (Elsevier) by publicly available macros and the SR-Accelerator translation tool.^19,20^ The full search strategies can be found in the appendices (**See appendix, Supplemental Digital Content 1**, which displays the full search strategy, https://links.lww.com/PRSGO/E500.) The searches were run on January 23, 2024, references exported to EndNote 20, and deduplicated using Deduklick.^21^ Upon import into Covidence, further suggested duplicates were reviewed manually.

Titles and abstracts of the search results were independently screened by 2 reviewers (P.P. and J.S.) in Covidence. Selected references were obtained in full text and independently assessed for eligibility by 2 reviewers (P.P. and J.S.) in Covidence. Disagreements over eligibility during title–abstract or full-text screening were resolved under the supervision of the study leaders (T.I. and E.A.K.).

### Study Selection and Eligibility Criteria

Studies published after 1980 were screened by title and abstract and included if they reported on adult patients undergoing postoncological head and neck reconstruction with either a QoL outcome and/or PROMs in the title or abstract. The remaining articles were assessed in full text based on the following additional eligibility criteria: (1) adults; (2) location of reconstruction: head and neck = maxillofacial, enoral (including mandibular, tongue, oral cavity), pharyngeal, laryngeal; (3) type of reconstruction: free flap; (4) defect due to oncological tumor resection; and (5) QoL outcome or PROM tool. Studies that (1) did not include a QoL outcome; (2) did not use free flaps; and (3) included orbital reconstruction independent of maxillectomy, skull base reconstruction, ear reconstruction, scalp reconstruction, or nose reconstruction were excluded.

### Data Extraction

The 354 articles included in this review were extracted using a data extraction sheet shared by 3 reviewers (P.P., J.S., M.P.) according to the following predetermined variables: author; year of publication; country in which the study was conducted; involvement of otorhinolaryngology/ear, nose, and throat surgery/head and neck surgery (HNS); maxillofacial surgery; plastic and reconstructive surgery (PRS); number of patients included; number of patients completing the questionnaire; primary tumor; location of reconstruction; flap used; and PROM tool used, including nonstandardized questionnaires and donor-site questionnaires. Nonvalidated tools and nonstandardized questionnaires were summarized into 1 category.

### Statistical Methods

Descriptive statistics were used to summarize the distribution of PROMs and types of flaps used in the reviewed studies. Continuous variables were reported as means with SDs or medians with interquartile ranges, depending on the data distribution. Categorical variables were summarized as frequencies and percentages. All statistical analyses were performed using the R statistical software (Version 4.3.2, The R Foundation for Statistical Computing, Vienna, Austria). Figures were created using Prism (GraphPad, Version 10.2.3).

## RESULTS

### Search Results

**Fig. 1.**
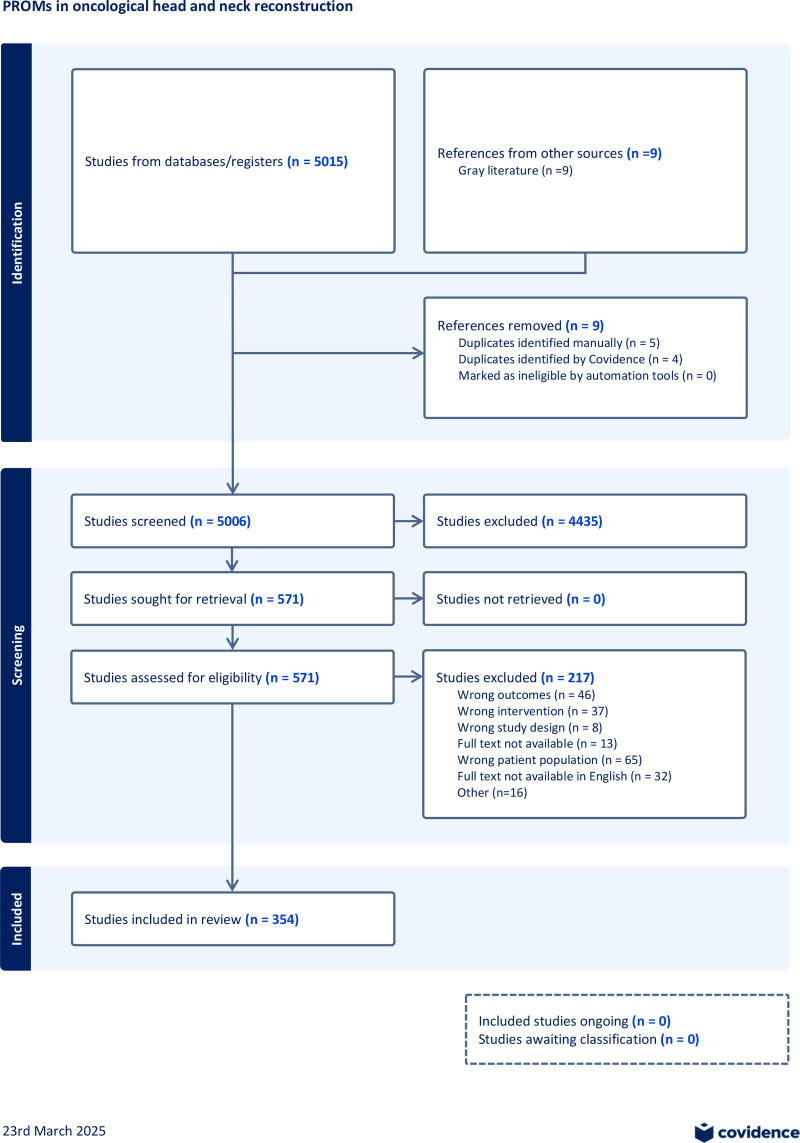
Preferred Reporting Items for Systematic Review and Meta-Analyses 2020 flow diagram for new systematic reviews, including searches of databases, registers, and other sources. Cochrane CENTRAL, the Cochrane Central Register of Controlled Trials.

**Fig. 2.**
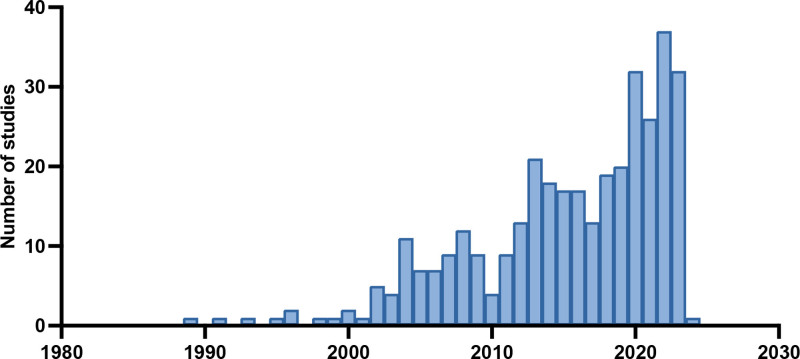
Studies by year of publication.

The initial electronic database search identified 8574 articles, of which 3599 were duplicates. The remaining 5006 studies were screened, yielding 571 articles for full-text assessment for eligibility. Of these 571 articles, 354 studies, involving a total of 30,369 patients, were included in the final review. A detailed review of the reasons for exclusion can be found in the screening segment of the Preferred Reporting Items for Systematic Reviews and Meta-Analyses flowchart (Fig. 1). We found an increasing trend of publications using these tools beginning in the year 1989 (Fig. 2). A variety of free flaps were used for reconstructive procedures, with the radial forearm free flap being used most often (Table 1). (**See table, Supplemental Digital Content 2**, which displays all used free flaps, https://links.lww.com/PRSGO/E503)

**Table 1.** Top 5 Free Flaps

Flaps	N Studies	% Studies	N Patients	% Patients
Radial forearm free flap	167	25.54	10,379	22.70
Fibula free flap	122	18.65	7186	15.72
Anterolateral thigh free flap	109	16.67	7195	15.74
Scapula free flap	43	6.57	3950	8.64
Latissimus dorsi free flap	31	4.74	4204	9.20

The table reflects multiple flap usage, with studies often reporting more than 1 flap.

Full Table Available in **Supplemental Digital Content 2,** https://links.lww.com/PRSGO/E503 https://links.lww.com/PRSGO/E500

### Most Commonly Used PROM Tools

A total of 94 different PROM tools were identified including the donor site-specific tools. **(See table, Supplemental Digital Content 3**, which displays all PROM tools. The table reflects multiple PROM tool usage, with studies often reporting more than 1 instrument, https://links.lww.com/PRSGO/E504.) The most commonly used PROM tool was the University of Washington Quality of Life (UW-QoL) Questionnaire version 4 (34.75%), followed by PROM assessment with a nonvalidated tool (25.99%). All nonvalidated tools were summarized into 1 category. The third most frequently used tool was the European Organisation for Research and Treatment of Cancer Quality of Life Questionnaire – Head and Neck Module 35 (EORTC QLQ-H&N35,22.32%). The 5 most frequently used tools are listed in Table 2. In total, 51.1% of studies used multiple PROM tools, whereas 48.9% used a single PROM tool.

**Table 2.** Five Most Frequently Used PROM Tools

PROM	N	%
UW-QoL Questionnaire version 4	123	34.75
Assessed patient-reported outcomes with nonvalidated tools	92	25.99
EORTC QLQ-H&N35	79	22.32
EORTC QLQ-C30	57	16.10
M. D. Anderson Dysphagia Inventory	20	5.65

The table reflects multiple PROM tool usage, with studies often reporting more than 1 instrument.

EORTC QLQ-C30, European Organisation for Research and Treatment of Cancer Quality of Life Questionnaire – Core 30.

### Interdisciplinary Collaboration and PROM Selection

**Fig. 3.**
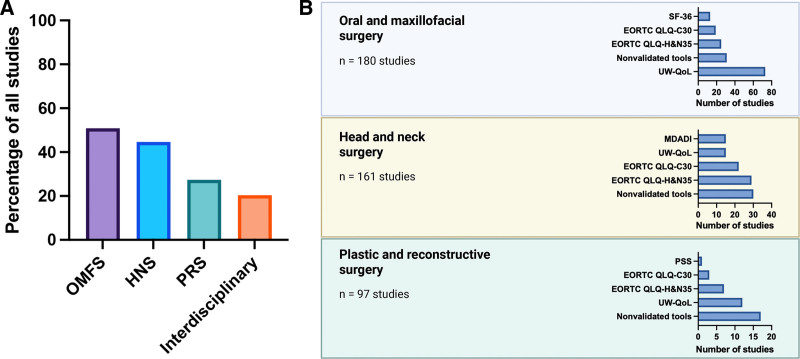
PROMs by surgical discipline. A, Percentage of involvement of surgical specialties related to all 354 studies. B, Top 5 PROMs by discipline. MDADI, M. D. Anderson Dysphagia Inventory; OMFS, oral and maxillofacial surgery; PSS, Performance Status Scale; SF-36, 36-Item Short Form Health Survey.

The majority of studies (79.66%, n = 282) were conducted within a single surgical discipline, whereas interdisciplinary collaboration was reported in only 20.34% of cases (n = 72). Across all studies, both single-discipline and interdisciplinary, oral and maxillofacial surgery was involved in 50.85% (n = 180), HNS in 45.48% (n = 161), and PRS in 27.40% (n = 97; Figure 3A).

Interdisciplinary studies (16.20%, n = 23) and oral and maxillofacial surgery–only publications (33.80%, n = 73) most frequently used the UW-QoL tool. PRS-only publications most commonly used nonvalidated tools (33.33%, n = 17), followed by the UW-QoL tool (23.53%, n = 12). Similarly, HNS-exclusive studies most frequently used nonvalidated tools (14.49%, n = 30), followed by the EORTC QLQ-H&N35 tool (14.01%, n = 29). The top 5 tools used by discipline are detailed in Table 3 and Figure 3B.

**Table 3.** Top 5 PROMs by Surgical Discipline

PROM	Interdisciplinary, N = 72		OMFS Exclusive, N = 145		HNS Exclusive, N = 98		PRS Exclusive, N = 45	
	n	%	n	%	n	%	n	%
UW-QoL	23	16.20	73	33.80	15	7.25	12	23.53
Nonvalidated tools	14	9.86	31	14.35	30	14.49	17	33.33
EORTC QLQ-H&N35	18	12.68	25	11.57	29	14.01	7	13.73
EORTC QLQ-C30	13	9.15	19	8.80	22	10.63	3	5.88
M. D. Anderson Dysphagia Inventory	4	2.82	1	0.46	15	7.25		

OMFS, Oral and maxillofacial surgery; PS, Plastic surgery.

### Geographic Differences in PROM Use

**Fig. 4.**
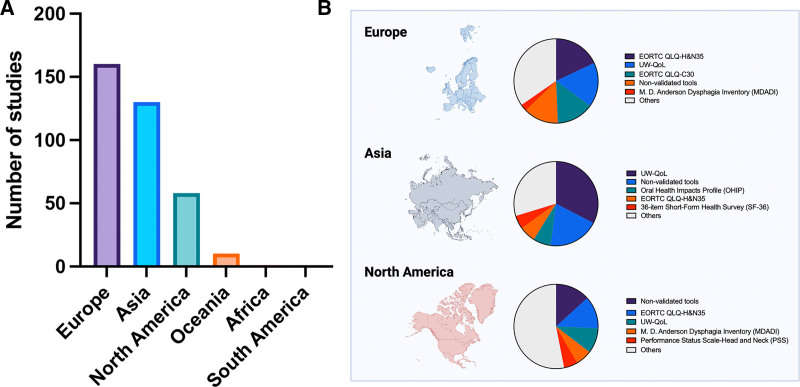
PROMs by geographical location. A, Number of studies published, allocated to respective continents. B, Top 5 PROMs by continent: Europe, Asia, and North America.

Across geographic regions, European research groups contributed the highest number of studies (n = 160), followed by those based in Asia (n = 130) and North America (n = 58) (Fig. 4A). Patterns of PROM use varied across these regions. Studies from North America demonstrated a relatively higher reliance on nonvalidated tools. In contrast, the UW-QoL was the most commonly used instrument in studies from Asia, whereas European studies used both the UW-QoL and the EORTC QLQ-H&N35 at comparable rates (Fig. 4B).

### Donor-site Outcomes

Donor site–specific PROMs were infrequently used, with only 22% (n = 45) of studies addressing these aspects comprehensively. In addition, 8.76% of these were nonvalidated tools created specifically for the respective studies. There was a variety of tools addressing donor sites from the upper and lower extremities, aesthetic aspects, scar evaluation, and pain-related outcomes. All tools used are listed in Table 4. In studies where PRS was involved, significantly more donor-site questionnaires were used (31.11% with PRS involved versus 15.53% in studies without PRS involvement, *P* = 0.019 using the Fisher exact test).

**Table 4.** All Donor-specific PROMs Used

PROM	N	% Overall	% Donor
Nonvalidated tools	31	8.76	49.21
Disabilities of Arm, Shoulder and Hand questionnaire	13	3.67	20.63
Ankle-Hindfoot Scoring System	8	2.26	12.70
Visual Analog Scale (aesthetic)	7	1.98	11.11
Lower Extremity Functional Scale	6	1.69	9.52
Patient and Observer Scar Assessment Scales	6	1.69	9.52
Lower Limb Core Scale	5	1.41	7.94
Vancouver Scar Scale	5	1.41	7.94
Morphological and functional self-assessment (adapted from Bozec)	5	1.41	7.94
Simple Shoulder Test	4	1.13	6.35
Foot and Ankle Disability Index	1	0.28	1.59
Pain Disability Questionnaire	1	0.28	1.59

## DISCUSSION

The use of PROMs in head and neck reconstruction was first reported by Freedlander et al^22^ in 1989. They assessed patients and their spouses regarding any embarrassment or difficulties the patients experienced related to their appearance, speech, and eating and drinking function.^22^ Additionally, patients completed 2 validated tools: the Hospital Anxiety and Depression Scale and the General Health Questionnaire.^22^ Following this pioneering work in implementing PROMs in head and neck reconstruction, a decade of merely sporadic use of these tools followed. In the meantime, as oncological therapies and survival rates for HNC patients have improved, the focus has expanded beyond disease-free survival to prioritizing the best possible QoL outcomes for patients. Our review confirms a steady increase in the use of PROMs in head and neck reconstruction. However, we identified significant heterogeneity, with 94 different tools in use, many of which are nonvalidated, hindering meaningful cross-study comparison. This contrasts with fields such as breast reconstruction, where the BREAST-Q has emerged as a widely adopted standard for both clinical care and research.^23^ A comparable extent of consensus does not exist for head and neck reconstruction.^24,25^

### Differences in PROM Tool Selection Based on Specialty

PROM selection varies by surgical specialty, reflecting differences in clinical priorities, focus areas, and patient populations. Table 5 summarizes the 3 most commonly used PROM tools overall. The UW-QOL questionnaire is widely used in HNC research.^26,27^ It consists of 12 domains assessing physical and social-emotional functions and is popular among oral and maxillofacial (OMF) and plastic surgeons due to its focus on oral function, appearance, and patient-prioritized outcomes.^28,29^ In contrast, head and neck surgeons frequently use a combination of the European Organisation for Research and Treatment of Cancer Quality of Life Questionnaire – Core 30 (EORTC QLQ-C30), which assesses general cancer-related QoL, and the EORTC QLQ-H&N35, reflecting the perspective of HNS specialists that extends beyond surgery. Head and neck surgeons, being involved in all stages of HNC care, may prefer these tools for cross-specialty evaluations and comparisons across cancer types.^30^ Furthermore, the EORTC QLQ-C30 and the EORTC QLQ-H&N35 were the most commonly used combination of assessment tools, appearing together in 52 studies. This was followed by combinations involving a nonvalidated tool with either the EORTC QLQ-H&N35 (14 studies) or the UW-QoL (13 studies). Other frequently used combinations included the EORTC QLQ-C30 with the UW-QoL, as well as the Oral Health Impact Profile with the UW-QoL (both combined in 10 studies each). The frequent use of these combinations reflects the popularity of the individual tools, which are also among the most commonly used stand-alone instruments. However, the reliance on multiple tools underscores the absence of a single, comprehensive instrument that addresses both general cancer-related QoL and HNC-specific issues, while also spanning the full breadth of surgical and medical treatment of HNC. A full list of the most commonly used PROM pair combinations can be found in the supplemental digital content. (**See table, Supplemental Digital Content 4**, which displays the most commonly used PROM pair combinations, https://links.lww.com/PRSGO/E505.)

**Table 5.** Comparison of the 3 Most Commonly Used PROM Tools

	UW-QOL	EORTC QLQ- C30	EORTC QLQ-H&N35
No. items	12	30	35
No. domains evaluated	12	16	18
Names of domains and scales evaluated	Pain, appearance, activity, recreation, deglutition, chewing, speech, shoulder, taste, saliva, mood, and anxiety. Includes a global QoL item and an importance rating (allowing patients to classify which domains are most important to them)	Five functional scales: physical performance, functional performance, emotional performance, cognitive performance, and social performance Three symptom scales: fatigue, pain, nausea, and vomiting One global health scale Six items of other symptoms: dyspnea, lack of appetite, insomnia, constipation, and diarrhea One scale of financial impact assessment	Pain, swallowing, cognitive problems, speech, eating in public, social contact, sexuality, dental problems, open mouth, dry mouth, sticky saliva, cough, malaise, consumption of analgesics, nutritional supplements, tube feeding, loss and gain of weight
Scale	0–100	0–100	0–100
Key advantages	Shorter and easier to score, widely validated, and translated into more than 30 languages. Popular among OMF surgeons due to its emphasis on oral functions, and among plastic surgeons for its inclusion of appearance-related domains and the opportunity for patients to specify which outcomes are most important to them. Plastic surgeons’ awareness of patient satisfaction as a central measure of the success of reconstructive surgery	Provides a broad assessment of cancer-related QoL, allowing for comparison across different cancer types. Most commonly used by ENT surgeons in combination with the EORTC QLQ-H&N35, as it facilitates cross-specialty and multidisciplinary evaluations	Offers a comprehensive evaluation of HNC-specific concerns, covering symptoms, treatment side effects, and social impact. Commonly used alongside the EORTC QLQ-C30 for a more detailed analysis of treatment success and morbidity, incorporating general cancer-related and HNC-specific aspects
Common users	OMF surgeons and plastic and reconstructive surgeons	ENT surgeons and multidisciplinary teams	ENT surgeons and multidisciplinary teams
Challenges	Lacks systemic cancer-related QoL assessment	Requires combination with QLQ-H&N35 for HNC-specific details	Needs EORTC QLQ-C30 for a complete QoL assessment

OMFS, Oral and maxillofacial surgery.

These inconsistencies may reflect how HNC reconstruction is distributed across specialties and healthcare systems. However, this variability impedes objective comparisons and limits broader progress in the field.^31^ It therefore seems intuitive that the variety in surgical specialties involved leads to inconsistencies in the use of evaluation tools. The lack of standardization in PROM tools for HNC reconstruction though hinders objective outcome comparisons and, as such, the further improvement of surgical results. Establishing standardized, validated PROMs could enable better cross-study comparisons; support clinical decision-making; and, in the end, improve patient QoL. Standardized, validated PROM use across disciplines and geographic regions could help build extensive databases, enabling cross-study and center comparisons, possibly supporting regulatory discussions.^30,32^ Using instruments with unverified validity wastes resources and may be unethical, burdening patients without yielding meaningful insights.^33–35^ Therefore, to achieve optimal patient outcomes through an interdisciplinary team approach, where oncological resections are performed without compromise and reconstructive efforts restore anatomical integrity, establishing a universally accepted tool should become a priority to effectively evaluate these treatment approaches.

### Geographic Differences in PROM Tool Selection

Geographic differences in PROM selection suggest that institutional practices, healthcare infrastructure, and prevailing surgical leadership influence the choice and implementation of outcome measures. For example, studies from North America, although fewer in number, relied more heavily on nonvalidated tools, indicating a potential gap in standardized assessment frameworks. In contrast, Asian studies more frequently used the UW-QoL questionnaire, whereas European groups showed a balanced use of both the UW-QoL and the EORTC QLQ-H&N35. It is important to note the geographic underrepresentation of studies from South America, Africa, and Oceania in our review. This highlights a need for increased research efforts and validation of PROM tools in these regions to ensure broader global applicability and inclusivity of patient-reported outcomes in head and neck reconstruction. It is worth considering that the availability of specific PROM tools varied across regions and over time, which may have influenced their regional uptake. For example, tools introduced earlier in certain regions may have been adopted more widely. Although our findings suggest such patterns, it remains uncertain whether these chronological differences have a causal relationship with PROM tool selection, and further investigation is warranted. The evolution of PROM tool use by year and geographic region can be found in the supplemental digital content. (**See figure, Supplemental Digital Content 5**, which displays the evolution of PROM tools by year and geographic region, https://links.lww.com/PRSGO/E506.) The geographic differences in PROM tool selection highlight the absence of a globally accepted standard and underscore the need for international consensus to enable reliable benchmarking and collaborative progress in head and neck reconstruction.

### Donor-site Evaluation

Donor-site outcomes remain underreported, with only 22% of studies addressing them. Furthermore, among these, most studies relied on nonvalidated tools specifically created for their respective investigations. Notably, studies involving plastic surgeons assessed donor morbidity nearly twice as often, suggesting a greater emphasis on this aspect of care within the specialty (31.11% versus 15.53%). This may be attributed to greater awareness among plastic and reconstructive surgeons of the overall impact of donor-site morbidity on outcomes and overall patient satisfaction. As such, many PROM tools developed within PRS for other anatomical areas, such as the BREAST-Q, routinely incorporate elements related to donor-site morbidity.

### Strengths and Limitations

To manage retrieval volume, a QoL/PROM search concept was included, which balances sensitivity and specificity, but may also limit the search. Additionally, we assumed that the lead authors’ specialty reflected the primary surgical discipline, which may not always hold true.

The strength of this study lies in its exceptionally comprehensive scope, spanning a time frame of 44 years and including data from 354 studies and more than 30,000 patients. To the best of our knowledge, this is the first study that evaluates the impact of geographic variations on PROM tool selection. By considering regional differences, we provide valuable insights into how cultural, healthcare system, and practice variations may influence the choice and application of PROMs in head and neck reconstruction.

## CONCLUSIONS

This systematic review highlighted substantial heterogeneity in the use of PROMs in microsurgical head and neck reconstruction, reflecting both geographic and disciplinary differences. Such variation limits standardized outcome assessment and highlights the need for improved interdisciplinary collaboration. The observed low number of interdisciplinary publications may suggest an area for further exploration, such as potential barriers to interdisciplinary collaboration, which warrants future investigation. To achieve standardization, each specialty must reaffirm its role through collaboration, and international efforts should focus on adopting a shared, validated PROM. This tool should capture functional, aesthetic, and donor-site outcomes to better reflect patient-centered care. Ultimately, improving how we measure what matters to patients will require consensus, collaboration, and a shared commitment to integrating patient perspectives into reconstructive success.

## DISCLOSURE

The authors have no financial interest to declare in relation to the content of this article.

## ACKNOWLEDGMENTS

A machine learning–based natural language processing model (large language model) was used for grammatical and spelling corrections. The authors are fully responsible for the originality, validity, and integrity of the content of their article.

## Supplementary Material


