# Supplementary material for: Patient-reported Outcome Measures in Head and Neck Reconstruction: A Systematic Review Across Disciplines and Geographical Locations
Source: Plast Reconstr Surg Glob Open. 2025 Dec 9;13(12):e7293. doi: 10.1097/GOX.0000000000007293 (PMC12688922; doi:10.1097/GOX.0000000000007293)
Supplement: Supplementary file 1 [file gox-13-e7293-s001.pdf]

Supplemental Digital Content 1: Full search strategy.

A systematic literature search was performed to identify articles on post-oncological head and neck reconstruction in adults, which reported a QoL outcome such as patient satisfaction or a PROM tool assessment. Search strategies for Embase (embase.com), Medline (Ovid), and the Web of Science Core Collection (webofscience.com) were composed by an information specialist (C.A.-H.). They comprised database-specific subject headings and text words covering the search concepts HNC, surgical flaps, and QoL/PROM. Names of known PROMs were omitted from the search string to prevent bias in retrieval. No restrictions on language or publication date were applied. Conference abstracts were excluded. The year 1980 was selected to ensure the inclusion of early foundational studies and to capture the full evolution of PROM development in head and neck reconstruction. Search syntax was translated from Embase-Elsevier by publicly available macros and by using the SR-accelerator translation tool.

Embase.com
(20240123; 3,429 hits)
('Head and neck tumor'/exp OR (((Head OR Heads OR Neck OR Necks OR HN OR 'upper aerodigestive tract*' OR UADT OR oesophag* OR esophag* OR face OR faces OR facial OR eye OR eyes OR eyelid* OR orbit OR orbital OR mouth* OR oral OR gingiva* OR lip OR lips OR palate* OR palatal OR salivary OR parotid OR 'sublingual gland*' OR 'submandibular gland*' OR tongue* OR Otorhinolaryng* OR Otolaryng* OR Oropharyng* OR ear OR ears OR auricle* OR auricular OR larynx OR laryngeal OR glottis OR epiglottis OR 'vocal chord*' OR 'vocal cord*' OR nose OR noses OR nasal OR sinus* OR sinonasal OR Pharynx OR pharyngeal OR Hypopharyn* OR Nasopharsyn* OR Oropharyn* OR Tonsil OR tonsillar OR Parathyroid* OR thyroid* OR trachea* OR airway* OR Cheek* OR bucca* OR Throat* OR gorge* OR skull* OR crani* OR viscerocrani* OR neurocrani* OR calvari* OR maxill* OR jaw* OR mandib* OR zygomatic* OR lacrim* OR palatin* OR vomer* OR scalp) NEAR/3 (carcino* OR adenocarcino* OR cancer* OR neoplas* OR tumor* OR tumour* OR malignan* OR dysplas* OR adenom* OR lymphadenom* OR polyp* OR melanom* OR papilloma* OR metastas* OR chondrosarcoma OR ameloblastoma)) OR HNSCC):ab,ti,kw)
AND
('surgical flaps'/exp OR 'tissue flap'/de OR 'adipofascial flap'/de OR 'anterolateral thigh flap'/de OR 'chimeric flap'/de OR 'deep inferior epigastric perforator flap'/de OR 'fasciocutaneous flap'/de OR 'gracilis flap'/de OR 'inferior gluteal artery perforator flap'/de OR 'inguinal flap'/de OR 'island flap'/de OR 'latissimus dorsi flap'/de OR 'muscle flap'/de OR 'myocutaneous flap'/de OR 'perforator flap'/de OR 'skin flap'/exp OR 'transverse rectus abdominis musculocutaneous flap'/de OR 'vertical rectus abdominis musculocutaneous flap'/de OR 'venous flap'/de OR 'omental flap'/de OR 'v y plasty'/de OR 'skin graft'/de OR 'full thickness skin graft'/de OR (((surgical OR free OR tissue OR adipofascial OR adipo-fascial OR 'anterolateral thigh' OR 'antero-lateral thigh' OR ALT OR 'medial thigh' OR 'posterior thigh' OR pudendal-thigh OR chimeric OR fasciocutaneous OR fascio-cutaneous OR gracilis OR IGAP OR I-GAP OR inguinal OR groin OR island OR muscle OR myocutaneous OR myo-cutaneous OR muscular-cutaneous OR musculocutaneous OR musculo-cutaneous OR perforator OR skin OR cutaneous OR dermal OR pedicle* OR bipedicle* OR transposition* OR Singapore OR VRAM OR gluteal OR gluteus OR omentum OR omental OR peritoneum OR peritoneal OR V-Y OR fillet OR local OR advancement OR 'fasciae latae' OR Martius) NEAR/3 (flap OR flaps OR graft OR grafts)) OR 'free inguinal graft' OR 'skin pedicle' OR 'flap repair' OR 'flap reconstruction*' OR 'V-Y plasty'):ab,ti,kw)
AND
('Quality of life'/exp OR 'quality of life assessment'/exp OR 'quality of working life'/exp OR 'quality of life questionnaire'/exp OR 'quality of life scale'/exp OR 'satisfaction'/exp OR 'daily life activity'/exp OR 'Personal autonomy'/exp OR 'wellbeing'/exp OR Happiness/exp OR Self-Concept/de OR 'body image'/exp OR 'Family Relation'/de OR 'functional status'/de OR 'health status'/de OR 'pain'/de OR 'headache and facial pain'/exp OR 'eye pain'/exp OR 'eyelid pain'/exp OR 'gingiva pain'/exp OR 'jaw

pain'/exp OR 'larynx pain'/exp OR 'mouth pain'/exp OR 'oropharynx pain'/exp OR 'salivary gland pain'/exp OR 'scalp pain'/exp OR 'tooth pain'/exp OR 'physical mobility'/de OR 'employment'/exp OR 'absenteeism'/de OR 'medical leave'/de OR 'sexuality'/exp OR 'Patient Reported Outcome'/de OR 'self report'/exp OR 'questionnaire'/exp OR 'assessment of humans'/exp OR 'patient reported outcomes measurement information system'/exp OR 'patient reported outcome measurement information system'/exp OR 'patient reported outcome measurement information system physical function'/exp OR 'patient reported outcome measurement information system score'/exp OR 'patient reported outcomes measurement information system 29'/exp OR 'patient reported outcome measure'/exp OR 'patient reported outcomes'/exp OR 'patient reported outcome measurement'/exp OR ((quality NEAR/3 life) OR hrql OR qol OR 'personal satisfaction' OR 'patient satisfaction' OR 'job satisfaction' OR 'life satisfaction' OR (daily NEAR/3 activ*) OR 'Personal autonomy' OR wellbeing OR well-being OR wellness OR Happiness OR (Patient* NEAR/3 (perception* OR experience* OR preference*)) OR 'fear of death' OR 'family relation*' OR 'positive experience' OR ((body OR self) NEXT/1 (image* OR concept OR awareness OR representation)) OR Selfconcept OR ((function* OR health OR physical*) NEAR/3 (status* OR outcome*)) OR pain* OR headache OR mobilit* OR employment OR unemployment OR absenteeism* OR ((medical OR sick) NEXT/1 leave) OR sexual* OR ((patient* OR self) NEAR/3 (report* OR perception OR rating)) OR ((Patient-Centered OR Patient-Centred OR Patient-Perceived OR Patient-Derived OR Patient-Based OR Patient-Rated) NEAR/5 (Outcome* OR Measure* OR Score* OR pain OR soreness OR ache OR hurt OR mobility OR 'internal rotation' OR motility OR flexibility OR 'range of motion' OR function* OR disability OR disabilities OR disablement* OR handicap OR impairment* OR assessment*)) OR PROM OR PROMs OR Survey* OR Questionnaire* OR scale*):ab,ti,kw)
NOT
(('animal'/de OR 'animal experiment'/exp OR 'nonhuman'/de) NOT ('human'/exp OR 'human experiment'/de))
NOT
[conference abstract]/lim

Medline
(Ovid MEDLINE(R) ALL 1946 to January 22, 2024; 2,978 hits)
(exp "Head and neck neoplasms"/ OR exp "Skull neoplasms"/ OR (((Head OR Heads OR Neck OR Necks OR HN OR upper aerodigestive tract* OR UADT OR oesophag* OR esophag* OR face OR faces OR facial OR eye OR eyes OR eyelid* OR orbit OR orbital OR mouth* OR oral OR gingiva* OR lip OR lips OR palate* OR palatal OR salivary OR parotid OR sublingual gland* OR submandibular gland* OR tongue* OR Otorhinolaryng* OR Otolaryng* OR Oropharyng* OR ear OR ears OR auricle* OR auricular OR larynx OR laryngeal OR glottis OR epiglottis OR vocal chord* OR vocal cord* OR nose OR noses OR nasal OR sinus* OR sinonasal OR Pharynx OR pharyngeal OR Hypopharyn* OR Nasopharsyn* OR Oropharyn* OR Tonsil OR tonsillar OR Parathyroid* OR thyroid* OR trachea* OR airway* OR Cheek* OR bucca* OR Throat* OR gorge* OR skull* OR crani* OR viscerocrani* OR neurocrani* OR calvari* OR maxill* OR jaw* OR mandib* OR zygomatic* OR lacrim* OR palatin* OR vomer* OR scalp) ADJ3 (carcino* OR adenocarcino* OR cancer* OR neoplas* OR tumor* OR tumour* OR malignan* OR dysplas* OR adenom* OR lymphadenom* OR polyp* OR melanom* OR papilloma* OR metastas* OR chondrosarcoma OR ameloblastoma)) OR HNSCC).ab,ti,kf.)
AND
(exp surgical flaps/ OR (((surgical OR free OR tissue OR adipofascial OR adipo-fascial OR anterolateral thigh OR antero-lateral thigh OR ALT OR medial thigh OR posterior thigh OR pudendal-thigh OR chimeric OR fasciocutaneous OR fascio-cutaneous OR gracilis OR IGAP OR I-GAP OR inguinal OR groin OR island OR muscle OR myocutaneous OR myo-cutaneous OR muscular-cutaneous OR musculocutaneous OR musculo-cutaneous OR perforator OR skin OR cutaneous OR dermal OR pedicle* OR bipedicle* OR transposition* OR Singapore OR VRAM OR gluteal OR gluteus OR omentum OR omental OR peritoneum OR peritoneal OR V-Y OR fillet OR local OR advancement OR

fasciae latae OR Martius) ADJ3 (flap OR flaps OR graft OR grafts)) OR free inguinal graft OR skin pedicle OR flap repair OR flap reconstruction* OR V-Y plasty).ab,ti,kf.)
AND
(exp Quality of Life/ OR functional status/ OR exp Health Status/ OR exp Body Image/ OR Pain/ OR Earache/ OR Eye Pain/ OR exp Facial Pain/ OR exp Headache/ OR Neck Pain/ OR mobility/ OR exp Employment/ OR Absenteeism/ OR Sick Leave/ OR exp Activities of Daily Living/ OR exp Sexuality/ OR exp Sexual Behavior/ OR exp "Surveys and Questionnaires"/ OR exp Patient Reported Outcome Measures/ OR exp Self Report/ OR ((quality ADJ3 life) OR hrql OR qol OR personal satisfaction OR patient satisfaction OR job satisfaction OR life satisfaction OR (daily ADJ3 activ*) OR Personal autonomy OR wellbeing OR well-being OR wellness OR Happiness OR (Patient* ADJ3 (perception* OR experience* OR preference*)) OR fear of death OR family relation* OR positive experience OR ((body OR self) ADJ (image* OR concept OR awareness OR representation)) OR Selfconcept OR ((function* OR health OR physical*) ADJ3 (status* OR outcome*)) OR pain* OR headache OR mobilit* OR employment OR unemployment OR absenteeism* OR ((medical OR sick) ADJ leave) OR sexual* OR ((patient* OR self) ADJ3 (report* OR perception OR rating)) OR ((Patient-Centered OR Patient-Centred OR Patient-Perceived OR Patient-Derived OR Patient-Based OR Patient-Rated) ADJ5 (Outcome* OR Measure* OR Score* OR pain OR soreness OR ache OR hurt OR mobility OR internal rotation OR motility OR flexibility OR range of motion OR function* OR disability OR disabilities OR disablement* OR handicap OR impairment* OR assessment*)) OR PROM OR PROMs OR Survey* OR Questionnaire* OR scale*).ab,ti,kf.)
NOT (exp animals/ NOT humans/)

Web of Science Core Collection
(20240123; Web of Science Core Collection, Editions = A&HCI , BKCI-SSH , BKCI-S , CCR-EXPANDED , ESCI , IC , CPCI-SSH , CPCI-S , SCI-EXPANDED , SSCI; 2,167 hits)
TS=(((Head OR Heads OR Neck OR Necks OR HN OR "upper aerodigestive tract*" OR UADT OR oesophag* OR esophag* OR face OR faces OR facial OR eye OR eyes OR eyelid* OR orbit OR orbital OR mouth* OR oral OR gingiva* OR lip OR lips OR palate* OR palatal OR salivary OR parotid OR "sublingual gland*" OR "submandibular gland*" OR tongue* OR Otorhinolaryng* OR Otolaryng* OR Oropharyng* OR ear OR ears OR auricle* OR auricular OR larynx OR laryngeal OR glottis OR epiglottis OR "vocal chord*" OR "vocal cord*" OR nose OR noses OR nasal OR sinus* OR sinonasal OR Pharynx OR pharyngeal OR Hypopharyn* OR Nasopharsyn* OR Oropharyn* OR Tonsil OR tonsillar OR Parathyroid* OR thyroid* OR trachea* OR airway* OR Cheek* OR bucca* OR Throat* OR gorge* OR skull* OR crani* OR viscerocrani* OR neurocrani* OR calvari* OR maxill* OR jaw* OR mandib* OR zygomatic* OR lacrim* OR palatin* OR vomer* OR scalp) NEAR/3 (carcino* OR adenocarcino* OR cancer* OR neoplas* OR tumor* OR tumour* OR malignan* OR dysplas* OR adenom* OR lymphadenom* OR polyp* OR melanom* OR papilloma* OR metastas* OR chondrosarcoma OR ameloblastoma)) OR HNSCC)
AND
TS=(((surgical OR free OR tissue OR adipofascial OR adipo-fascial OR "anterolateral thigh" OR "antero-lateral thigh" OR ALT OR "medial thigh" OR "posterior thigh" OR pudendal-thigh OR chimeric OR fasciocutaneous OR fascio-cutaneous OR gracilis OR IGAP OR I-GAP OR inguinal OR groin OR island OR muscle OR myocutaneous OR myo-cutaneous OR muscular-cutaneous OR musculocutaneous OR musculo-cutaneous OR perforator OR skin OR cutaneous OR dermal OR pedicle* OR bipedicle* OR transposition* OR Singapore OR VRAM OR gluteal OR gluteus OR omentum OR omental OR peritoneum OR peritoneal OR V-Y OR fillet OR local OR advancement OR "fasciae latae" OR Martius) NEAR/3 (flap OR flaps OR graft OR grafts)) OR "free inguinal graft" OR "skin pedicle" OR "flap repair" OR "flap reconstruction*" OR "V-Y plasty")
AND
TS=((quality NEAR/3 life) OR hrql OR qol OR "personal satisfaction" OR "patient satisfaction" OR "job satisfaction" OR "life satisfaction" OR (daily NEAR/3 activ*) OR "Personal autonomy" OR wellbeing OR

well-being OR wellness OR Happiness OR (Patient* NEAR/3 (perception* OR experience* OR preference*)) OR "fear of death" OR "family relation*" OR "positive experience" OR ((body OR self) NEAR/0 (image* OR concept OR awareness OR representation)) OR Selfconcept OR ((function* OR health OR physical*) NEAR/3 (status* OR outcome*)) OR pain* OR headache OR mobilit* OR employment OR unemployment OR absenteeism* OR ((medical OR sick) NEAR/0 leave) OR sexual* OR ((patient* OR self) NEAR/3 (report* OR perception OR rating)) OR ((Patient-Centered OR Patient-Centred OR Patient-Perceived OR Patient-Derived OR Patient-Based OR Patient-Rated) NEAR/5 (Outcome* OR Measure* OR Score* OR pain OR soreness OR ache OR hurt OR mobility OR "internal rotation" OR motility OR flexibility OR "range of motion" OR function* OR disability OR disabilities OR disablement* OR handicap OR impairment* OR assessment*)) OR PROM OR PROMs OR Survey* OR Questionnaire* OR scale*)
NOT DT=(Meeting Abstract)
