# Supplementary material for: Patient-reported Outcome Measures in Head and Neck Reconstruction: A Systematic Review Across Disciplines and Geographical Locations
Source: Plast Reconstr Surg Glob Open. 2025 Dec 9;13(12):e7293. doi: 10.1097/GOX.0000000000007293 (PMC12688922; doi:10.1097/GOX.0000000000007293)
Supplement: Supplementary file 2 [file gox-13-e7293-s002.pdf]

Supplemental Digital Content 2

Flaps	N Studies	% Studies	N Patients	% Patients
RFFF (radial)	167	25.54	10379	22.70
FFF (fibula)	122	18.65	7186	15.72
ALT FF (anterolateral thigh)	109	16.67	7195	15.74
SFF (scapula)	43	6.57	3950	8.64
Latissimus dorsi FF	31	4.74	4204	9.20
Rectus abdominis FF	28	4.28	2095	4.58
Deep circumflex iliac artery flap (DCIA FF)	22	3.36	1558	3.41
Jejunal FF	18	2.75	921	2.01
Lateral upper arm FF	16	2.45	994	2.17
iliac crest FF/ ilium flap	15	2.29	863	1.89
Pectoral FF	14	2.14	2454	5.37
Ulnar Forearm Free Flap (UFFF)	11	1.68	875	1.91
Serratus FF	5	0.76	458	1.00
Deep inferior epigastric artey perforator (DIEP) FF	4	0.61	126	0.28
Medial Sural Artery Perforator Free Flap (MSAP)	4	0.61	231	0.51
Temporal FF	4	0.61	396	0.87
Thoracodorsal artery perforator FF (TDAP)	4	0.61	124	0.27
AMT FF (anteromedial thigh)	3	0.46	132	0.29
Peroneal FF (without bone)	3	0.46	138	0.30
Sternocleidomastoid/ sternomastoid clavicluar FF	3	0.46	130	0.28
Vastus Lateralis Myofascial Free Flap (VLM FF)	3	0.46	60	0.13
Ileocolic FF	2	0.31	46	0.10
Large intestine FF	2	0.31	49	0.11
Lateral thigh FF (LTF)	2	0.31	70	0.15
Lateral upper arm flap	2	0.31	129	0.28
Medial upper arm FF	2	0.31	58	0.13
Platysma FF	2	0.31	215	0.47
Posterior brachial FF	2	0.31	88	0.19

Flaps	N Studies	% Studies	N Patients	% Patients
Profunda Artery Perforator FF	2	0.31	135	0.30
Thoracodorsal artery perforator FF (TAP FF)	2	0.31	57	0.12
Cheek muscles FF	1	0.15	63	0.14
Double paddle peroneal artery perforator FF (DPAP FF)	1	0.15	57	0.12
Gracilis FF	1	0.15	96	0.21
Medial femoral condyle FF	1	0.15	30	0.07
SIEA FF	1	0.15	48	0.10
Soleus perforator flap	1	0.15	82	0.18
Supraclavicular artery island flap (SCAIF)	1	0.15	24	0.05

Supplemental Digital Content 2: All used free flaps. Table reflects multiple flap usage, with studies often reporting more than one flap. FF = free flap.
