# Supplementary material for: Patient-reported Outcome Measures in Head and Neck Reconstruction: A Systematic Review Across Disciplines and Geographical Locations
Source: Plast Reconstr Surg Glob Open. 2025 Dec 9;13(12):e7293. doi: 10.1097/GOX.0000000000007293 (PMC12688922; doi:10.1097/GOX.0000000000007293)
Supplement: Supplementary file 3 [file gox-13-e7293-s003.pdf]

Supplemental Digital Content 3

PROM	N	%
University of Washington-Quality of Life Questionnaire version 4	123	34.75
Assessed patient reported outcomes with non-validated tools	92	25.99
EORTC QLQ-H&N35	79	22.32
EORTC QLQ-C30	57	16.10
M. D. Anderson Dysphagia Inventory (MDADI)	20	5.65
Oral Health Impacts Profile (OHIP)	18	5.08
Performance Status Scale-Head and Neck (PSS)	18	5.08
36-item Short-Form Health Survey (SF-36)	17	4.80
Voice Handicap Index (VHI)	12	3.39
Hospital Anxiety and Depression Scale (HADS)	11	3.11
Speech Handicap Index (SHI)	11	3.11
Eating Assessment Tool-10 scores (EAT-10)	8	2.26
Patient Concerns Inventory (PCI)	8	2.26
FACE-Q module	7	1.98
Functional Assessment of Cancer Therapy-Head and Neck (FACT-HN)	7	1.98
Voice-Related Quality of Life Measure (V-RQOL)	6	1.69
Functional Oral Intake Scale (FOIS)	5	1.41
Karnofsky Performance Status Scale (KPSS)	5	1.41
Swallowing Quality of Life index (SWAL-QOL)	5	1.41
Dysphagia Handicap Index (DHI)	4	1.13
Dysphagia Outcomes and Severity Scale (DOSS)	4	1.13
EORTC-QLQ-HN43	4	1.13
Functional intraoral Glasgow scale (FIGS)	4	1.13
RAND 36-item Health Survey (RAND36)	4	1.13
VAS pain score	4	1.13
Denture satisfaction index (DSI)	3	0.85
Distress thermometer survey instrument	3	0.85

PROM	N	%
EuroQol visual analogue scale (EQ-5D-5L)	3	0.85
Functional Assessment Cancer Therapy General Scale (FACT-G)	3	0.85
Functional Living Index-Cancer (FLIC)	3	0.85
Therapy Outcome Measure Dysphonia Scale (Table 2)	3	0.85
Deglutition Handicap Index (DHI)	2	0.56
GOHAI (Global Oral Health Assessment Index) questionnaire	2	0.56
General Anxiety Disorder questionnaire (GAD)	2	0.56
MOS 8-Item ShortForm Health Survey (SF-8)	2	0.56
Macmillan Distress Thermometer (pictorial mood assessment tool)	2	0.56
Mental Health Composite Score (MCS)	2	0.56
Obturator Functioning Scale	2	0.56
Patient Health Questionnaire (PHQ)	2	0.56
Physical health Composite Score (PCS)	2	0.56
Short Form-12 version1 (SF-12v1)	2	0.56
Sydney Swallow Questionnaire (SSQ scale)	2	0.56
Therapy Outcome Measure Dysphagia Scale (TOM)	2	0.56
WHO-QoL BREF	2	0.56
Berlin questionnaire (risk for obstructive sleep apnea)	1	0.28
Bochum Patient Questionnaire on Rehabilitation	1	0.28
Body Satisfaction Scale (BSS)	1	0.28
Cambridge Plastic Surgery Postoperative Evaluation Scale	1	0.28
Decision Regret Scale (DRS)	1	0.28
Derriford Appearance Scale Short Form (DAS24)	1	0.28
EORTC QLQ-ELD14	1	0.28
Functional Assessment of Chronic Illness Therapy Fatigue (FACIT-F)	1	0.28
Functional outcome swallowing scale (FOSS)	1	0.28
Hirose Standard for speech intelligibility	1	0.28
Human figure test	1	0.28
IMAGE-HN	1	0.28

PROM	N	%
Impact on Participation and Autonomy (IPA)	1	0.28
Insomnia Severity Index	1	0.28
Inventory to Measure and Assess imaGe disturbancE–Head & Neck (IMAGE-HN)	1	0.28
Linear Analogue Self Assessment method (LASA, 1 item version)	1	0.28
Liverpool oral rehabilitation questionnaire (LORQ) v3	1	0.28
MD Anderson Symptom Inventory for head and neck cancer (MDASI-HN)	1	0.28
Mann Assessment of Swallowing Ability- Oral Cancer (MASA-OC)	1	0.28
Mini Mental Adjustment to Cancer (MINIMAC)	1	0.28
Modified Cornell Medical Index (+ 2 questions (about convenience and psychosocial))	1	0.28
Montgomery Asberg Depression Rating Scale (MADRS)	1	0.28
Neck Disability Index (NDI)	1	0.28
Oral symptom check list	1	0.28
Posttraumatic Growth Inventory (PTGI)	1	0.28
Quality Of Recovery 40 (QOR40) questionnaire	1	0.28
Short Musculoskeletal Function Assessment	1	0.28
Standardized University of Michigan Head and Neck Specific Quality of Life questionnaire	1	0.28
Supportive Care Needs Survey Short-Form 34 (SCNS-SF34)	1	0.28
Velopharyngeal Insufficiency Quality of Life (VPIQL) questionnaire	1	0.28
Visual Analogue Scale xerostomia questionnaire (VAS)	1	0.28
Visual analogue cosmesis score (0-100)	1	0.28
Work Ability Index (WAI)	1	0.28
World Health Organization Quality-of-Life	1	0.28
amyotrophic lateral sclerosis scale (ALSS)	1	0.28
disability assessment scale (WHO DAS II)	1	0.28
modified Godin Leisure-Time Exercise Questionnaire (GLTEQ)	1	0.28
revised Edmonton Symptom Assessment System (ESAS-r)	1	0.28
swallowing outcomes after laryngectomy (SOAL) questionnaire	1	0.28

Supplemental Digital Content 3: All used PROM tools. Table reflects multiple PROM tool usage, with studies often reporting more than one instrument.
