# Supplementary material for: Patient-reported Outcome Measures in Head and Neck Reconstruction: A Systematic Review Across Disciplines and Geographical Locations
Source: Plast Reconstr Surg Glob Open. 2025 Dec 9;13(12):e7293. doi: 10.1097/GOX.0000000000007293 (PMC12688922; doi:10.1097/GOX.0000000000007293)
Supplement: Supplementary file 4 [file gox-13-e7293-s004.pdf]

Supplemental Digital Content 4

PROM pairs	N
EORTC QLQ-C30 | EORTC QLQ-H&N35	52
Assessed patient reported outcomes with non-validated tools | EORTC QLQ-H&N35	14
Assessed patient reported outcomes with non-validated tools | University of Washington-Quality of Life Questionnaire version 4	13
EORTC QLQ-C30 | University of Washington-Quality of Life Questionnaire version 4	10
Oral Health Impacts Profile (OHIP) | University of Washington-Quality of Life Questionnaire version 4	10
EORTC QLQ-H&N35 | University of Washington-Quality of Life Questionnaire version 4	9
36-item Short-Form Health Survey (SF-36) | University of Washington-Quality of Life Questionnaire version 4	7
Assessed patient reported outcomes with non-validated tools | EORTC QLQ-C30	7
Assessed patient reported outcomes with non-validated tools | Performance Status Scale-Head and Neck (PSS)	7
M. D. Anderson Dysphagia Inventory (MDADI) | Speech Handicap Index (SHI)	7

Supplemental Digital Content 4: Most commonly used PROM pair combinations.
