# Supplementary material for: Patient-reported Outcome Measures in Head and Neck Reconstruction: A Systematic Review Across Disciplines and Geographical Locations
Source: Plast Reconstr Surg Glob Open. 2025 Dec 9;13(12):e7293. doi: 10.1097/GOX.0000000000007293 (PMC12688922; doi:10.1097/GOX.0000000000007293)
Supplement: Supplementary file 5 [file gox-13-e7293-s005.pdf]

Supplemental Digital Content 5

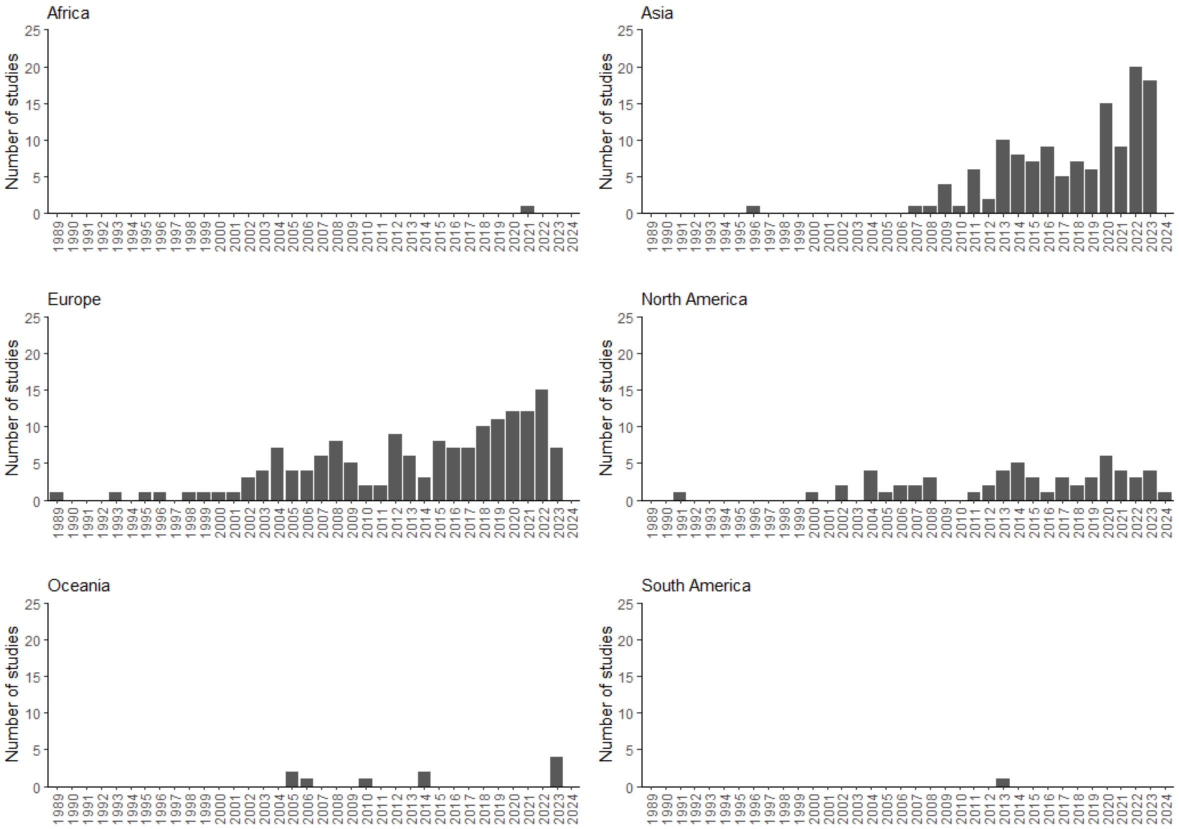

Supplemental Digital Content 5: Evolution of PROM tools by year and geographic region.
